# Tungiasis in the Sanumás Amerindians in the Amazon Rainforest, Brazil: Prevalence, Intensity and Morbidity

**DOI:** 10.3390/tropicalmed8080386

**Published:** 2023-07-28

**Authors:** Yago Ranniere Teixeira Santana, Lucas Felipe Carvalho Oliveira, Gabriela Mafra Lima, Renata Velôzo Timbó, Eliane Mateus Pires, Amanda Ramos de Brito, Ana Carolina Tardin Martins, Vivyanne Santiago Magalhães, Ana Carolina Mota de Faria, Ada Amalia Ayala Urdapilleta, Isabelle Roger, Rafael Rocha de Andrade, Luciana Pereira Freire Martins, Marcos Pellegrini, Fabiola Christian Almeida de Carvalho, David Dias Araújo, Daniel Holanda Barroso, Carina Nogueira Garcia, Hermann Feldmeier, Ciro Martins Gomes

**Affiliations:** 1 Secretaria Especial de Saúde Indígena (SESAI), Ministério da Saúde do Brasil, Brasília 70.910-900, Brazil; yagorts92@gmail.com (Y.R.T.S.); lucasfelipe250@hotmail.com (L.F.C.O.); bio.elianepires@gmail.com (E.M.P.); 2 Programa de Pós-Graduação em Saúde Coletiva, Universidade de Brasília, Brasília 70.910-900, Brazil; 3 Distrito Sanitário Especial Indígena (DSEI) Yanomami, Boa Vista 69.301-08, Brazil; gabrielamafralima@gmail.com; 4 Programa de Pós-Graduação em Ciências Médicas, Universidade de Brasília, Brasília 70.910-900, Brazil; renata.timbo@gmail.com (R.V.T.); caroltardin@gmail.com (A.C.T.M.); irogercongo@gmail.com (I.R.); rafaelandrade@unb.br (R.R.d.A.); danielhbarroso@gmail.com (D.H.B.); carina.n.garcia@gmail.com (C.N.G.); 5 Programa de Pós-Graduação em Ciências da Saúde, Universidade Federal de Roraima, Boa Vista 69.310-000, Brazil; aramosbrito@outlook.com (A.R.d.B.); fabiola.carvalho@ufrr.br (F.C.A.d.C.); 6 Secretaria de Vigilância em Saúde, do Ministério da Saúde do Brasil, Brasília 70.723-040, Brazil; vivyanne.magalhaes@saude.gov.br (V.S.M.); ana.faria@saude.gov.br (A.C.M.d.F.); 7 Secretaria de Saúde do Distrito Federal, Brasília 70.719-040, Brazil; ada.urdapilleta@gmail.com; 8 Programa de Pós-Graduação em Patologia Molecular, Universidade de Brasília, Brasília 70.910-900, Brazil; 9 Dimensuri Assessoria Técnica, Brasília 70.391-900, Brazil; daviddiasaraujo@gmail.com; 10 Institute of Microbiology, Infectious Diseases and Immunology, Charité—University Medicine Berlin, D-14195 Berlin, Germany; feldmeier.fuberlin@t-online.de

**Keywords:** tungiasis, Amerindian people, One Health, neglected diseases

## Abstract

Background: Tungiasis is a disease associated with extreme poverty. We aimed to evaluate the prevalence of tungiasis in six different settlements of the Sanumás indigenous community in a remote area in the Auaris region, Yanomami territory, Brazil. Methods: We conducted an observational study to detect clinical and epidemiological factors associated with tungiasis using a cross-sectional strategy and multivariate logistic regression. Soil analysis was performed by visual and microscopic methods. Results: We examined 555 persons, 45 of whom had active tungiasis; 18 cases were classified as mild, 16 as moderate and 11 as severe. The disease was significantly more prevalent in children than in adults (odds ratio (OR) 15.77; 95% confidence interval (CI) = 5.34–67.91; *p* < 0.001). Soil infestation was significantly related to the occurrence of human tungiasis (OR = 12.29; 95% CI = 3.75–45.88). The sex and GPS location of the houses were not related to the occurrence of tungiasis. Conclusions: We conclude that tungiasis is an important problem in the Sanumás community, especially for children. We suggest that interruption of the off-host transmission cycle, together with regular treatment [human and animal interventions], must be prioritized to achieve control of tungiasis in indigenous populations.

## 1. Introduction

**Figure 1:**
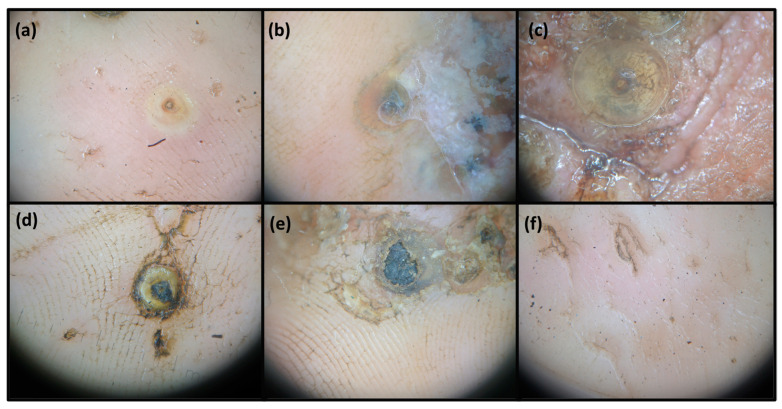
Dermoscopic representation (10×) of the natural history of *Tunga penetrans* in humans according to the Fortaleza classification. (**a**) The penetration phase; (**b**) the phase of beginning hypertrophy; (**c**) the white halo phase; (**d**) the involution phase; (**e**) residues in the host’s skin; and (**f**) scars from healed infection. Images were generated using a Nikon 1 J2 camera (Nikon, Tokyo, Japan) and a Dermlite 2 Hybrid dermoscope (Dermlite, San Juan Capistrano, CA, USA).

Tungiasis (sand flea disease) is a parasitic skin disease caused by the female sand flea *Tunga penetrans* (Siphonaptera), which is native to South America [1]. It is a zoonotic neglected disease widespread in resource-poor settings in South America and sub-Saharan Africa [2,3]. The female penetrates the skin, eventually reaching the size of a pea. Through an opening in the skin of 250 µm, which represents the rear abdominal cone, oxygen is taken up, fertilization occurs, eggs are expelled and fecal material is excreted. According to the Fortaleza classification, the on-host development of *T. penetrans* is stratified into five stages (Figure 1) [4].

The epidemiology of tungiasis in South America is poorly known. In Brazil, the disease mainly affects locations with less social and economic development in the northern and northeastern regions of the country [5]. According to a recent systematic review of the literature, the prevalence of tungiasis in Brazil ranged from 1.6% to 54.8%, predominantly affecting school age children [5]. No relevant data on tungiasis in the northern region of Brazil, where the Amazon rainforest is located, were found [5].

In Brazil, similar to what is described for other endemic areas, most cases are of limited disease with a small number of lesions [6]. Although tungiasis is usually a self-limited infection, in endemic areas, lesions and morbidity accumulate over time, eventually leading to mobility impairment and a deteriorated health status, particularly in Amerindians [7,8,9]. Close, home or peridomestic contact with pets, such as cats and dogs, was more related to the higher frequency of tungiasis than the presence of other domestic animals, such as pigs, chickens, horses or sheep [5].

The objective of this study was to determine the prevalence, intensity and morbidity of tungiasis in six settlements of the Sanumás community, Roraima State, Brazil. 

## 2. Materials and Methods

### 2.1. Study Area and Population

The present study is part of a larger Brazilian government project aimed at the control of tungiasis in indigenous communities in Brazil [10]. This first access to the Yanomami territory, a remote Amazonian area, was important for the recognition of the problem and for projecting future actions. 

The Yanomami territory is a protected region with an area of 192,000 km^2^ located west of the Guiana Massif in Brazil and Venezuela, South America. The Auaris territory and the Sanumás community are near the upper effluents on the right margin of the Rio Branco. The Sanumás community is divided into small settlements formed by families living in 2 to 40 houses. Reportedly, the Sanumás have suffered from tungiasis for more than 20 years [11]. The Sanumás comprise a subgroup of the Yanomami linguistic family [12]. The Sanumás have a very close relationship with pets, mainly dogs, that are used for hunting. Dogs serve as hosts for *T. penetrans*.

**Figure 2:**
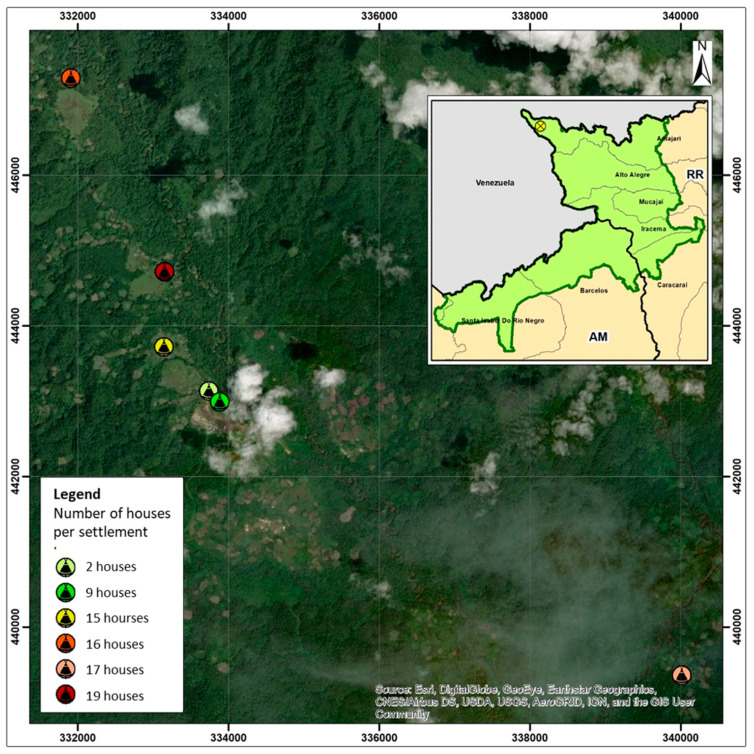
Satellite view of the Auaris communities. Access point: (03°00′106″ N 064°29′364″ W). Map generated by ArcGis version 10 environment (Esri, Reddlands, CA, USA).

We examined all the inhabitants living in the six Sanumás settlements (Karanaú, Kulapoipú, Psicultura, Katarrinha, Caixa D’Água and Kiripassipu) previously found to have the highest tungiasis prevalence according to administrative secondary data from the Secretaria Especial de Saúde Indígena (SESAI), Brazilian Ministry of Health [13,14,15]. The Sanumás live in the extreme north of the Yanomami territory, distributed in a 55-square-kilometer area (Access point: (4°00′1″ N 64°29′22″ W; altitude = 759 m above sea level) [10,11,12]. High-resolution spatial analysis was used to identify the different Auaris settlements (Dimensuri Assessoria Técnica, Brasília, Brazil) (Figure 2). Each household was geomapped (mobile phone Mi 10t PRO global version; Xiaomi, Beijing, China) (Figure 2). 

### 2.2. Examination of Humans and Animals

Examination was performed from 26 January to 5 February 2022 by a team comprising a dermatologist (CMG), an entomologist (AF) and a local indigenous health agent (interpreter). This is considered the dry season in the region and is probably related to greater soil infestation. There are no data proving a higher incidence of tungiasis in this period. The evaluation of each patient lasted from 2 to 10 min and each house was evaluated for an approximate period of 30 min. Tungiasis was diagnosed clinically, as previously described [13]. The whole body was examined [16]. Tungiasis severity was classified as follows: 1 = mild tungiasis (<10 active lesions, restricted to the feet); 2 = moderate tungiasis (<10 active lesions on the feet + hands); and 3 = severe tungiasis (>10 lesions on the feet, hands and other topographic areas). The presence of tungiasis in domestic animals was examined by veterinarians (VSM and RRA). Veterinarians examined animals that were gently restrained by their owners. The evaluation comprised a complete veterinary clinical consultation and included the identification of tungiasis lesions and the documentation of itching, drowsiness and prostration.

### 2.3. Living Conditions

The team knew in advance that none of the houses in the settlements had basic sanitation or rooms and that all the houses were built on compacted natural soil. The construction material of the houses was always similar, made from plant material or clay. The presence of walls in the houses was evaluated to quantify easier access to the internal regions of the houses by domestic animals.

Houses were numbered to facilitate future location. The house number was computed according to the proximity to the main access to the community (river or tracks). Smaller numbers of houses can represent easier access for domestic animals, mainly dogs, proven to be involved in the occurrence of tungiasis according to previous research [5]. Our team observed that the dogs circulate daily between the different settlements in search of food or accompanying their owners. The distance between the houses and to the access points could not be measured due to the difficulty generated by geographical features, such as mountains, valleys and canyons.

### 2.4. Examination of Soil

The parasites were recovered from the soil of the houses as they jumped onto white sheets of A4 paper after scarifying the adjacent soil. This method was only effective for recovering adult fleas. The first sheet of paper was placed next to the fire, usually in the center of the house, where off-host stages of *T. penetrans* are expected to occur. The leaves were then placed 1.5 m apart from each other, occupying the largest internal distance between the limits of the houses, resulting in 3 to 5 leaves per house. After 3 min, the entomologist performed a visual count using a Dermlite 2 Hybrid dermoscope (Dermlite, San Juan Capistrano, CA, USA). The infestation was quantified as follows: 0 = no infestation (no sheet with adult fleas), 1 = light infestation (only 1 sheet with adult fleas), 2 = medium infestation (2 sheets with adult fleas), and 3 = severe infestation (3 or more sheets with parasites). When any one of the sheets had more than 5 fleas captured, the classification was increased by 1 point up to a maximum of 3 (severe infestation). This method had been used by governmental institutions of Brazil and Colombia and to our knowledge had not been previously validated. Microscopic analysis of each 30 g soil sample was performed at the Dermatology Laboratory, University of Brasília, Brazil, for correct microscopic identification of the collected adult fleas and for the identification of eggs deposited in the soil, following previous publications on taxonomy [17].

### 2.5. Statistical Analysis

We compared the demographic and clinical characteristics of patients diagnosed with tungiasis to those of patients without tungiasis (considered the control group) to identify factors associated with the occurrence of the disease [18]. Univariate comparisons of proportions were performed using the chi-square test or Fisher’s exact test, if appropriate. Regarding age, patients were divided into only two groups: children and adults. Exact age information was not reliable because of the absence of an official birth registration policy in this population. We performed the Wilcoxon matched pairs signed rank test to compare ordinal variables between the two groups.

After bivariate analysis, a multivariate logistic regression model was constructed. The variables included in the model were chosen according to clinical relevance decided by a consensus formed by two specialized dermatologists (DB and CG). The variables, including cutoffs for odds ratio (OR) calculations [18], were as follows: sex; house number (≥4); house soil infestation according to the direct detection method (=3 or not); number of houses in the community (≥17); number of inhabitants at the same house (≥10); and number and species of domestic animals living in the house (≥1).

Statistical analysis was performed using the program R, version 4.1.2 (R Core Team 2021; R Foundation for Statistical Computing, Vienna, Austria. URL https://www.R-project.org/. Logistic regression was performed using the glm and/or_glm functions and the “stats” and “oddsratio” packages, respectively (Schratz, P. (2017): R package ‘oddsratio’: Odds ratio calculation for GAM(M)s and GLM(M)s, version: 1.0.2, doi: 10.5281/zenodo.1095472). Statistical significance was defined as a *p* value < 0.05.

### 2.6. Ethics

Written informed consent was obtained from all subjects involved in the study or their legal guardians. The study was approved by the Ethics Committee of the Faculty of Medicine (UnB), by the Comissão Nacional de Ética em Pesqusisa (CONEP) (Reference code CAAE: 30638920.0.0000.0008) and by the Conselho Distrital de Saúde Indígena (CONDISI). Access by the research team to the Yanomami territory was provided by the Brazilian Ministry of Health.

## 3. Results

### 3.1. Demographic, Environmental and Sanitation Characteristics

**Figure 3:**
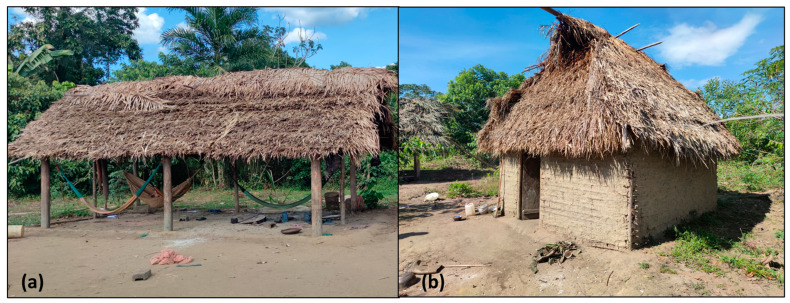
(**a**) House with no walls at all; (**b**) house with walls made from clay and sticks.

**Figure 4:**
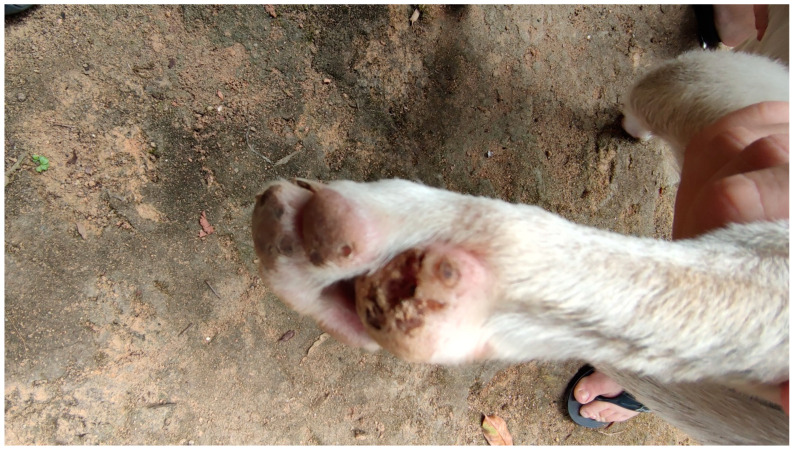
Footpad of a dog with several embedded sand fleas.

We examined 555 individuals living in 78 houses. The number of inhabitants living in each house ranged from 2 to 16. Demographic characteristics are summarized in Table 1. None of the houses were internally divided by walls. All the houses were built in a square rectangular format, ranging from 5 to 9 square meters (Figure 3). Fifty-four of the seventy-eight (69.23%) houses contained walls made of clay on all sides and a door made of wood, but five (6.41%) had at least one side without a wall, and nineteen (24.36%) had no walls (Figure 3). Children comprised 50.45% of the examined individuals. The proportion of males was higher than that of females: 53.15 and 46.85%, respectively. We examined 79 dogs, and 68 were found to be infected with *T. penetrans* (Figure 4). No other domestic animals were present.

### 3.2. Prevalence and Univariate Analysis

Forty-five (8.11%; 95% CI = 6.04–10.78%) individuals had tungiasis (Table 1). Nineteen houses (24.36%) were inhabited by patients diagnosed with active tungiasis. The prevalence of tungiasis was higher in children than in adults (15.00% versus 1.09%, respectively (*p* < 0.001)). Tungiasis was two times more prevalent in males (31 = 10.51%) than in females (14 = 5.38%) (*p* = 0.040). The number of persons living in the same house was larger for patients with tungiasis (median = 10; IQR = 4.00) than for those without tungiasis (median = 8; IQR = 4.00) (*p* < 0.001). Patients with tungiasis lived in houses classified by smaller numbers (median = 4; IQR = 10.00) than those without tungiasis (median = 8; IQR = 8.75) (*p* = 0.013), meaning that patients with tungiasis usually lived closer to the community access point than those without tungiasis (*p* < 0.001) (Table 1). The number of dogs living in each house, infected or not, was not related to the occurrence of tungiasis (*p* > 0.05).

### 3.3. Intensity and Morbidity

**Figure 5:**
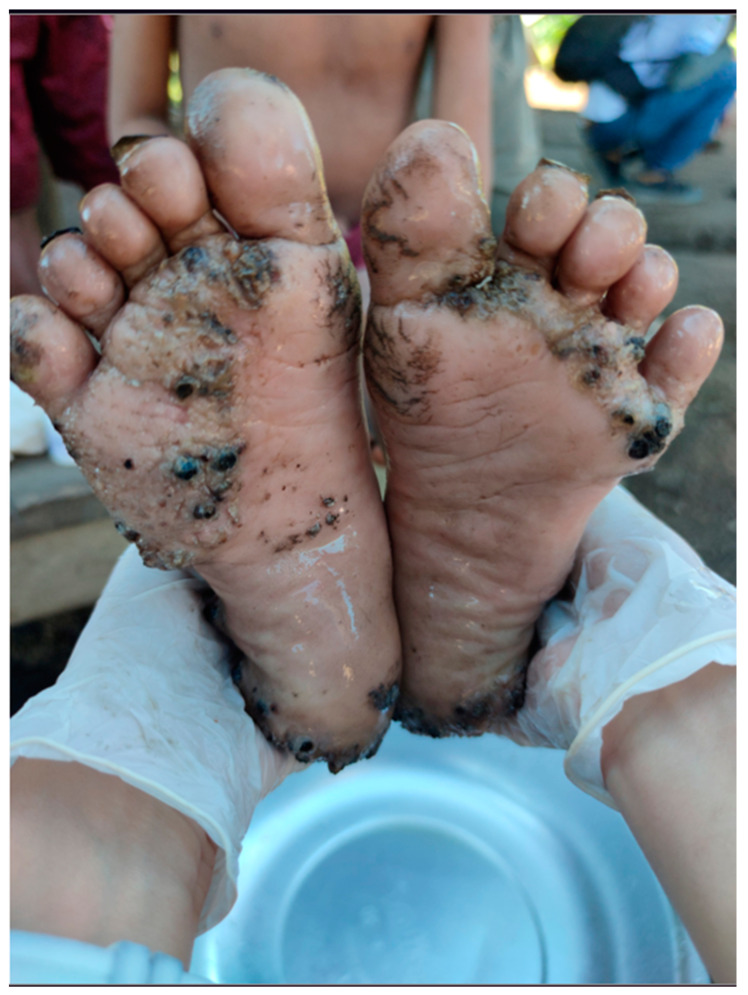
Tungiasis lesions in the interdigital areas and at the sole.

**Figure 6:**
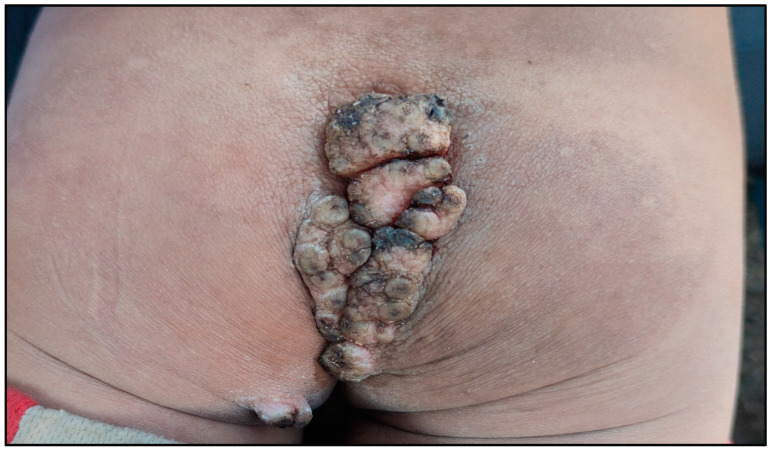
Coccygeal tungiasis.

Of the 45 individuals with tungiasis, 18 were classified as mild, 16 as moderate and 11 as severe. All patients had plantar lesions (Figure 5), while 18 also had associated hand lesions, and 11 also had perianal, coccygeal lesions. All patients who presented with perianal/coccygeal lesions also had palmar and plantar lesions (Figure 6).

### 3.4. Multivariate Analysis

The multivariate analysis showed that tungiasis occurred significantly more frequently in children than in adults (OR = 15.77; 95% CI = 5.34–67.91; *p* < 0.001) and that the severity of soil infestation classified as grade three was significantly related to the occurrence of tungiasis in humans (OR = 12.29; 95% CI = 3.75–45.88; *p* < 0.001) (Table 2). The number of houses in the community and the number of inhabitants living in the same house were associated with the presence of tungiasis, but the confidence intervals were rather wide (Table 2).

## 4. Discussion

Tungiasis is a neglected disease associated with extreme poverty [6,19,20]. It is also prevalent in environments with low socioeconomic development, including densely populated areas with poor living conditions, refugee camps and indigenous communities [5]. Children and elderly people are at higher risk of the disease and its complications [21]. To our knowledge, this is the first study on prevalence and morbidity in an indigenous population living in the Amazon rainforest of Brazil. The area is accessible only by plane and the health care infrastructure is rudimentary. Patients are dispersed over an area of 55 km^2^ (Figure 2).

Conducting clinical research in such a remote and isolated area represents a major scientific challenge. However, all vulnerable populations must be monitored for better provision of health services. The research team faced several challenges and went through some risks including regional air transport, river transport and displacement through dense forest areas. All this displacement was carried out with the support of the Brazilian Ministry of Health and the Brazilian Army. Clinical examinations and interviews were carried out inside the patients’ homes or in designated buildings and community meeting areas. Sometimes the professionals had close contact with the ground. The use of protective equipment was always reinforced. Lifeguards for river travel, sun protection clothing and rain protection equipment were available. Only one researcher acquired a single tungiasis lesion on the sole of the foot. Other occurrences were not reported by the research team.

The age distribution of the Sanumás population showed that 50.45% of the inhabitants were children. According to the multivariate analysis, children were significantly more frequently affected by tungiasis than adults (OR = 15.77; 95% CI = 5.34–67.91; *p* < 0.001). The high prevalence of tungiasis in children has been reported in other endemic areas in South America and Africa [9,21,22] and is explained by behavioral factors, such as unprotected contact with infested soils during play. However, immunological aspects must not be excluded [9,23]. Soil infestation was significantly related to the occurrence of human tungiasis at the household level (OR = 12.29; 95% CI = 3.75–45.88; *p* < 0.001).

The results of our multivariate analysis showed that soil infestation is a very good predictor for the occurrence of tungiasis at the household level. We therefore suggest that in situations such as in the Sanumás community, where transmission is supposed to occur indoors, treatment of infected humans and animals should be combined with interventions reducing the propagation of off-host stages in the soil. We did not observe different behavior or the use of different clothes according to gender. This may explain the lack of statistical relationship between sex and the occurrence of tungiasis.

The clinical characteristics of tungiasis in this indigenous population differ from those previously described in other Brazilian populations [9]. The high frequency of coccygeal lesions is the result of behavioral habits that consist of different activities in which children sit on the ground with little clothing protection. This is not common in more urbanized areas. Tungiasis control at the community level is complex, and in Brazil, it has never been attempted in a systematic manner. Experience in Africa suggests that prevention is possible through the use of repellents, distribution of shoes and construction of cemented floors [24,25,26]. Immediate control measures include the treatment of affected people with NYDA^R^, a formula of two dimeticone oils with low viscosity used in cases warranting treatment of active lesions. At the same time, the establishment of community measures to control infestations in homes, such as moistening floors and removing organic matter from the compound, are mandatory to avoid reinfection. The application of residual pyrethroid insecticides [27] and the treatment of dogs, pigs and other domestic animals have been considered appropriate additive control measures [28].

## 5. Conclusions

We conclude that tungiasis is an important health problem in the Sanumás community. The population living in the evaluated settlements is particularly vulnerable to tungiasis due to behavioral factors associated with the lack of basic sanitation and poor housing conditions. Control of tungiasis in Sanumás communities requires a One Health approach.

## Figures and Tables

**Table 1:** Sociodemographic characteristics of the study population.

Variable	Tungiasis Cases n (%) or Median (IQR)	Number of Persons Examined
Sex		
Female	14 (5.38%)	260
Male	31 (10.51%)	295
Age group		
Adult	3 (1.09%)	275
Children	42 (15.00%)	280
Median house location number	4.00 (10.00)	--
Median number of households in the community	17.00 (2.00)	--
Median number of inhabitants per house	10.00 (4.00)	--
Median severity of soil infestation in the patient’s home	3.00 (1.00)	--
Median number of dogs belonging to the household	1.00 (1.00)	--

Legend: n = number of patients; IQR = interquartile range.

**Table 2:** Multivariate analysis of factors associated with the presence of tungiasis.

Independent Variable	Odds Ratio (95% CI)	*p* Value
Age group (Children)	15.77 (5.34–67.91)	<0.001
Male sex	1.68 (0.80–3.66)	0.177
House location number classification (≥ 4)	0.92 (0.68–1.25)	0.610
Severity of soil infestation (=3 or not)	12.29 (3.75–45.88)	<0.001
Number of houses in the community (≥17)	90.83 (9.26–2140.87)	0.001
Number of inhabitants in the same house (≥10)	13.03 (2.00–102.58)	0.010
Number of dogs in the household (≥1)	0.73 (0.51–0.96)	0.048

Legend: CI = confidence interval.

## Data Availability

The data presented in this study are available on request from the corresponding author.
